# Supplementary material for: MetaRibo-Seq measures translation in microbiomes
Source: Nat Commun. 2020 Jun 29;11:3268. doi: 10.1038/s41467-020-17081-z (PMC7324362; doi:10.1038/s41467-020-17081-z)
Supplement: Supplementary file 10 — Supplementary Data 7 [file 41467_2020_17081_MOESM10_ESM.zip › File2/Confidence_High_Taxonomy/200867_out.krona.html]

Javascript must be enabled to view this page.

members
magnitude
magnitudeUnassigned
count
unassigned
taxon
rank

200867\_out

61

superkingdom
2
61

phylum
61
976

200643
61
class

order
171549
61

815
2
family

genus
2
816


SRS013098\_contig\_number\_29417
338188
1
species

1739298
1

SRS053398\_contig\_number\_679
species

family
59
171552

59
838
genus

species
59823
8

SRS019910\_contig\_number\_3584SRS049712\_contig\_number\_8745SRS050752\_contig\_number\_2950SRS077641\_contig\_number\_7605SRS077991\_contig\_number\_130SRS103987\_contig\_number\_189SRS1041031\_contig\_number\_29397SRS144603\_contig\_number\_12326

species

SRS078176\_contig\_number\_767
2293125
1

species
165179
50

SRS012969\_contig\_number\_14661SRS012989\_contig\_number\_8631SRS013521\_contig\_number\_4745SRS013638\_contig\_number\_10804SRS013687\_contig\_number\_27648SRS014415\_contig\_number\_1780SRS015590\_contig\_number\_16544SRS015782\_contig\_number\_38993SRS015794\_contig\_number\_2873SRS016541\_contig\_number\_17945SRS017307\_contig\_number\_30668SRS017375\_contig\_number\_52SRS018888\_contig\_number\_4253SRS019285\_contig\_number\_contig-100\_150.151SRS023526\_contig\_number\_36857SRS023914\_contig\_number\_16020SRS024132\_contig\_number\_16670SRS043841\_contig\_number\_10602SRS044535\_contig\_number\_14225SRS045195\_contig\_number\_848SRS045713\_contig\_number\_16297SRS046502\_contig\_number\_13727SRS047044\_contig\_number\_13084SRS047433\_contig\_number\_21832SRS049959\_contig\_number\_13367SRS049995\_contig\_number\_116SRS054059\_contig\_number\_416SRS064757\_contig\_number\_23165SRS065397\_contig\_number\_13662SRS074586\_contig\_number\_4849SRS077194\_contig\_number\_6280SRS077849\_contig\_number\_25798SRS078419\_contig\_number\_24207SRS097958\_contig\_number\_18284SRS104036\_contig\_number\_7059SRS1041134\_contig\_number\_14292SRS1041137\_contig\_number\_17328SRS1041140\_contig\_number\_13698SRS104327\_contig\_number\_6305SRS105082\_contig\_number\_350SRS143181\_contig\_number\_10114SRS143876\_contig\_number\_895SRS144297\_contig\_number\_contig-100\_172.90581SRS144506\_contig\_number\_42200SRS144537\_contig\_number\_35347SRS144714\_contig\_number\_621SRS146764\_contig\_number\_25889SRS147614\_contig\_number\_3481SRS148253\_contig\_number\_12591SRS150029\_contig\_number\_12225
